# Toward integrative approaches to study the causal role of neural oscillations via transcranial electrical stimulation

**DOI:** 10.1038/s41467-021-22468-7

**Published:** 2021-04-14

**Authors:** Valeriia Beliaeva, Iurii Savvateev, Valerio Zerbi, Rafael Polania

**Affiliations:** 1 Department of Health Sciences and Technology, ETH Zurich, Zurich, Switzerland; 2 Neuroscience Center Zurich, Switzerland, Zurich, Switzerland

**Keywords:** Electrophysiology, Neural circuits

## Abstract

Diverse transcranial electrical stimulation (tES) techniques have recently been developed to elucidate the role of neural oscillations, but critically, it remains questionable whether neural entrainment genuinely occurs and is causally related to the resulting behavior. Here, we provide a perspective on an emerging integrative research program across systems, species, theoretical and experimental frameworks to elucidate the potential of tES to induce neural entrainment. We argue that such an integrative agenda is a requirement to establish tES as a tool to test the causal role of neural oscillations and highlight critical issues that should be considered when adopting a translational approach.

## Abstract (web summary)

Transcranial electrical stimulation techniques may enable exploration of the role of neural oscillations in the brain. Here, the authors provide a perspective on its potential across systems, species, theoretical and experimental scales.

## Introduction

For more than a century, researchers have observed and studied rhythmic patterns in the brains of humans and other animals. However, it remains unclear whether these rhythms are essential for neural computations and the resulting behavior to occur, or whether their presence has no functional role. While some researchers support the idea that neural oscillations play a fundamental role in supporting efficient communication in the brain^1^, others suggest that information contained in such rhythmic patterns might be meaningless^2,3^. The latter group argue that signals recorded from a neural population aggregate the activity from various regions of the brain including the ones which are distant from the recording site^3^. Considering that geometry of the cells also affects the recorded signal, oscillations might contain mass of information that is hard to link to a particular brain source and establish its relationship with the observed behavior.

To resolve this debate, experimenters require techniques that modulate oscillatory dynamics of the targeted brain region or network to provide evidence for the involvement of a particular neural oscillation in the hypothesized cognitive process. Such assessments can be performed invasively via intracranial electrical stimulation or optogenetic techniques^4,5^. However, these methods cannot be applied in a routine fashion in humans, and therefore non-invasive and safe neuromodulation methods are required. Over the last two decades, methods belonging to the family of transcranial electrical stimulation (tES) were introduced with the potential to study the causal relationship between neural oscillations and behavior in humans in relatively safe and painless manner^6^.

One such approach, which was initially proposed to modulate neural oscillations, consists of the application of low-intensity alternating currents (tACS) to target brain structures in a frequency-specific manner^7^. Theoretically speaking, tACS has the potential to establish a causal link between the oscillatory pattern (modulated or induced) at the targeted brain structure and the resulting behavior, this given the assumption that tACS genuinely affects neural entrainment. Examples of its application include testing the causal role of specific frequency bands on cognitive^8^, sleep^9^, and motor functions^10^. Furthermore, tACS has been recently proposed as a tool to study how oscillatory coherence between spatially distinct nodes of functional networks contributes to behavior^8,11^. Some of these studies were criticized due to low degree of focality when using standard stimulation setup, but recent modeling work provides hints of how focality can be improved based on novel electrode montages^12,13^.

One issue with these approaches is that they cannot focally target areas in subcortical structures, thus limiting their potential applications. Recently developed methods based on multi-electrode tES protocols (such as temporal interference (TI) stimulation^14^) are promising technologies proposed to induce oscillatory entrainment of neural activity in subcortical structures without recruiting neurons of the overlying cortex^15^. While this method appears to overcome the constraint that only superficial structures may be focally affected, further studies are required to gain a more detailed understanding on the efficacy of these methods, their mechanism of action, and evaluate the relevance of this approach to study human cognition and behavior.

This line of research has led to controversial and heated discussions as to whether existing and emerging tES methodologies genuinely induce neural entrainment over targeted brain structures^16,17^. First, there is skepticism regarding the assumed direct effect of tES on target brain regions given that experimental evidence suggests that entrainment could occur via indirect stimulation of afferent nerves^18^, including retinal stimulation^19^. Second, current intensities conventionally applied in human studies might not be sufficient to genuinely induce neural entrainment^20,21^. Third, apparent neural entrainment might be the result of other neuromodulatory mechanisms such as excitability changes^22,23^ or increased burstiness of neurons^24^ in addition to (or instead of) genuine modulation of neural dynamics. Alongside these controversies, with the sharp increase of tES studies in the last years, there is some evidence that the impact of this methodology might not be fully reliable, potentially due to high variability of the induced effects across studies^25,26^.

As the field continues its maturation process, tES research is starting to evolve from hypothesis testing solely based on behavioral observations in humans towards a translational and integrative approach in order to elucidate the fundamental mechanisms of action of the technique. This means that researchers should adopt a multilevel research program including: (i) a deep understanding of neurophysiological principles underlying tES, (ii) connecting the principles of tES action to mechanistic theories of neural oscillations, and (iii) establishing a link between these neuro-mechanistic principles and the observed behavior. While the study across these different levels of abstraction is a common approach in different subfields of neuroscience^27^, it is just an emerging agenda in tES research. Some of these recent efforts include a multimethod and multidisciplinary approach that integrates computational modeling research with experiments in vitro, in vivo, and ex vivo (e.g., in rodents^14,21,28^ and monkeys^22,29^), as well as studies in clinical populations^11,30,31^. Here we argue that building links between the information gained at each of these different levels of abstraction is the natural next step in the maturation process of the tES technique.

While we consider this program to be essential in advancing our understanding of the effects induced by tES, these investigations must take into account the advantages and disadvantages that come with such a translational approach. For instance, translation of neuromodulatory interventions might be effective in a given system, species, or behavioral state but not in a different one. This challenge is not unique to brain stimulation research but is also present in other translational efforts, such as pharmacological interventions during drug discovery research. As we discuss in more detail below, these translational issues might be related to differences in neural circuits that are often assumed to underly the same behavioral function, a critical aspect often overseen in tES translational applications.

In this Perspective article, we advocate for these emerging and much needed joint translational efforts to elucidate whether existing and new tES methodologies can indeed be used to genuinely induce neural entrainment, and if so, whether these methods can be applied to test the causal role of neural oscillations in a routine fashion in humans. In order to discuss the potential advantages of this approach in more detail, this article is divided into three parts. First, we discuss critical aspects that need to be considered if one aims to translate knowledge of neuromodulation across species. In the following section, we propose that the effects induced by tES in any given system should be studied by evaluating the influences of the stimulation at different levels of information processing, and crucially establish paradigms that allow building mechanistic links across these levels. In the last part of the article, we illustrate how state-of-the-art computational models and individualized stimulation parameters may serve as methodologies for generating accurate predictions of the stimulation effects, thus improving the reliability of causal associations between neural oscillations and behavior.

Here, we focus on methods of the tES family that have the potential of modulating neural oscillations both in cortical and subcortical structures (e.g., tACS, TI, intersectional pulsed stimulation (ISP)^21^), as recently, these methods have sparked heated debates about their efficacy for inducing neural entrainment. Nevertheless, the views and opinions shared in this article might be well applicable to other forms of neuromodulation such as transcranial magnetic stimulation (TMS), a methodology that appears to effectively induce neural entrainment^32^, and transcranial focused ultrasound stimulation (tFUS)^33^. The goal of this article is to provide a perspective and not a detailed review of the literature. The impact of tES on neurophysiology and cognition, as well as methodological and theoretical aspects of neural entrainment have been reviewed in more detail elsewhere^6,34–37^.

## Comparative application of tES across species

Given the preservation of brain rhythms across systems and species alongside their tagged functional roles, it is tempting to translate directly the knowledge gained about the mechanisms of action induced by tES from one species to another^38^. However, we argue that this translation of knowledge across species requires careful considerations, in particular those related to differences in both anatomy and organization of functional networks. In this section, we attempt to bring awareness about some of the challenges that experimenters may need to consider when translating knowledge of targeted neuromodulatory interventions across species.

The ultimate goal of animal experiments is to have a meaningful predictive relevance for clinical applications, or predictive validity. For this purpose, animal studies with a translational goal in mind must be designed with consideration for their construct validity (the extent to which conclusions derived from the experiments in animals can be transferred to humans) and face validity (the degree to which an assessment or test subjectively appears to measure the variable or construct that it is supposed to measure). In tES studies, these concepts can be generalized in two major points. First, the delivery and distribution of electric fields/currents in animal models must correctly emulate those applied in human studies and have a similar interaction with the tissue micro-structure. Second, these electric fields should be designed to affect human-equivalent network-level targets, so that the responses/outcomes can be directly used to draw relevant inferences applied to humans both in health and disease. Each of these points requires further discussion.

### Comparable strength of the induced electric field

**Fig. 1:**
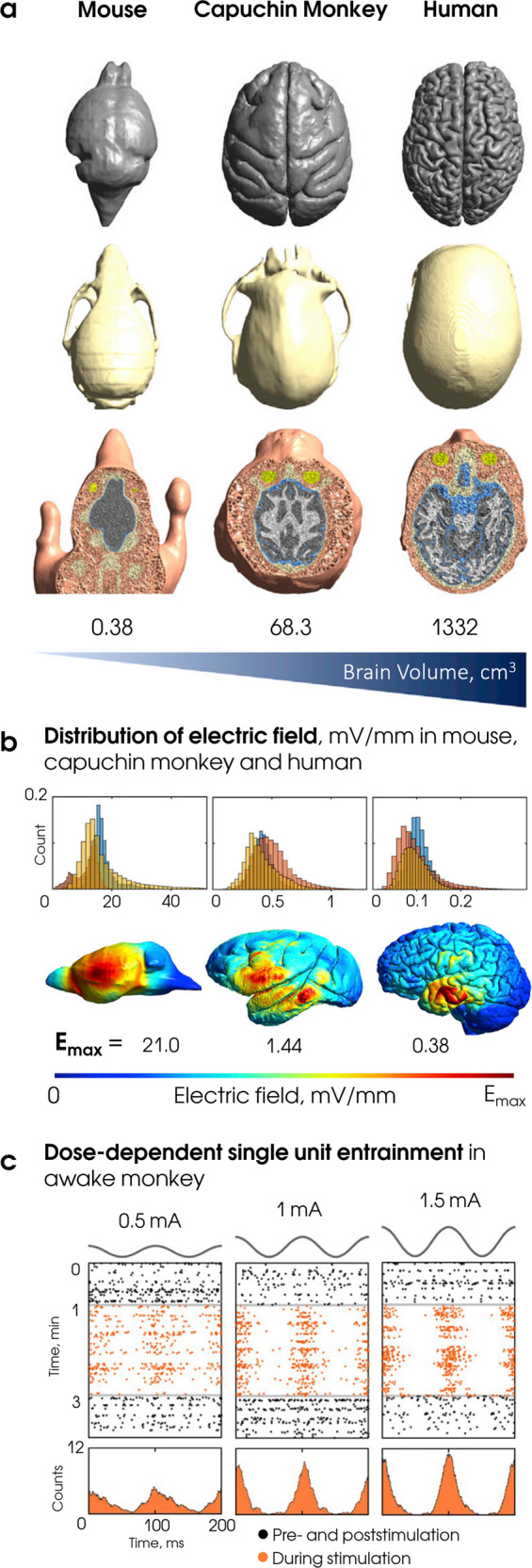
Comparison of the effects triggered by electrical stimulation in mouse, monkey, and human head models. Predictive models built for different species are a valuable tool for translational studies. Thanks to these models, researchers can develop a comparable stimulation setups across species. This was demonstrated in a recent study^40^, where electric fields estimated in the finite-element method (FEM) models were compared in mouse, monkey, and human head models. **a** Top row demonstrates lissencephalic brain of a mouse and gyrencephalic brains of a monkey and human. The presence of gyri affects the direction of the electric field, therefore electric fields in the human brain are better captured in monkeys than in mice; middle row—skull surfaces; third row—horizontal cut of FEM models. **b** Upper row demonstrates the distribution of the electric fields (mV/mm) in the brain with three different electrode setups (shown in red, blue, and yellow). Lower row—spatial distribution of the electric fields for the same electrode montage (left–right temporal areas) and stimulation intensity 1 mA with the maximum values for each model. The center of the distribution and the maximum field dramatically decreases from mouse to human with the increase in the head size under the comparable experimental setup. **c** The raster plots demonstrate the spike recordings from one representative neuron in neocortex of awake monkey before, during and after transcranial alternating current stimulation (tACS) at intensities 0.5, 1, and 1.5 mA peak-to-baseline. During stimulation (orange) spikes cluster around the stimulation peak, while in prestimulation and poststimulation periods (black) spikes are uniformly distributed. The spike rate increases with stimulation intensity, meaning that the level of entrainment depends on the dose of the non-invasively applied current. Panels **a** and **b** are adapted from ref. ^40^ with permission from Elsevier. Panel **c** is adapted from ref. ^24^. CC BY 4.0 (https://creativecommons.org/licenses/by/4.0/).

The first consideration relates to the obvious differences in brain anatomy and geometry of animal models compared to humans, and how this affects the electric field distributions in the brain. Most tES protocols produce a complex pattern of current flows, which result in electric fields and current densities that vary significantly across brain regions. In initial studies, current densities used in animal experiments were usually determined from estimations of human modeling work and it was assumed that the distribution of electric fields generated in the brain is ‘quasi-uniform’ across an area of the brain or even the entire brain/tissue^12,39^. However, it would be imprudent to adapt the parameters from human studies (and vice versa) assuming a linear relationship between brain volume and the amount of current required. These considerations are especially important for those brains that lack cortical circumvolutions and are much smaller in size, for example in rodents (Fig. 1a). In a recent study, investigators compared electric fields in head models of a mouse, non-human primate and human (Fig. 1b), finding that in each translational combination, different stimulation parameters must be appropriately adjusted in order to match electric fields within a given target region of interest across species^40^. Additionally, in the same study, they demonstrated that a primate model is better than a mouse model for the investigation of the mechanisms underlying electrical stimulation. Thus, differences across species of this kind change the effectiveness and variability of the results on physiology and behavior and limit direct translation to humans.

Another important aspect that complicates the direct comparison between human and animal studies is the interaction between electric field generated in the brain and the three-dimensional organization of neuronal compartments^41^. Electrical currents affect (de)hyperpolarization of all neuron subtypes, but the tES-induced effect depends on the orientation between the applied field and the soma-dendritic axis^31,36,42^.

Human and animal brain models can have significant differences in density and geometry of the neurons, types and distribution of ion channels within them, position of dendrites and axons relative to the electric field, degree of axon myelination or glial density; all these factors can severely affect the peak values and distribution of electric fields generated by tES^12^. The effectiveness of tES can be further complicated by the different contribution on current spread from skin, subcutaneous soft tissue, skull, and cerebrospinal fluid (CSF)^43^, as well as the behavioral state (in vivo, anesthetized, or ex vivo). For instance, studies in anesthetized rodents suggest that electric fields <1 V/m induced by tACS might not be sufficient to genuinely induce neural entrainment^21^, and critically if it occurs, it could be driven indirectly via the activation of afferent nerves^18^. In addition, evidence for inefficiency of low-intensity tACS came from a study with patients in awake and sleeping states, where oscillations recorded with intracranial EEG were not entrained during stimulation^20^. However, a recent seminal work found that tACS can genuinely induce neural entrainment in awake monkeys, crucially with electric fields <0.5 V/m and ~0.5 mA current intensities, which can be safely applied in humans using conventional protocols^24^ (Fig. 1c). These results are further supported by previous studies—also conducted in awake monkeys—that found neural entrainment in subcortical structures (however, this time applying higher current intensities)^22^. Crucially, a set of control experiments in these studies allowed to conclude that these results could not be caused by peripheral nerve stimulation^23^. In general, these findings suggest that brains from fully awake organisms are more susceptible to neural entrainment based on exogenously induced electric fields in the sub-V/m and sub-mA ranges, though the full support for this conclusion is complicated by the aforementioned study which demonstrated the absence of entrainment in the awake patients. Thus, future investigations should study whether the susceptibility to tES is higher in awake states in comparison with the anaesthetized conditions. Furthermore, the possibility of tES-induced peripheral nerve and retina stimulation reported in previous studies^18,19^ needs to be addressed whenever possible via additional control experiments.

Another aspect potentially contributing to the cross-species differences in tES experiments is the variation in morphology and electrical properties of the individual neurons in humans in comparison to mice and macaques. It was shown that human dendrites are longer and have higher branch complexity, leading to swifter attenuation of the external electrical inputs^44^. Also, in vitro studies of human cortical synapses exhibit up to four times faster synaptic depression recovery, lower action potential thresholds and faster firing rates, leading to higher information bandwidth in comparison to rodents. These findings highlight not just various differences in the electrical properties of neurons between humans and other mammals, but also point to the different operational modes of the homologous circuits^45^. Interestingly, variations in neuronal morphology in humans correlate with differences in performance in cognitive tests (e.g., IQ tests)^46^.

Whilst identification and effective control of all factors contributing to variability and mismatch between human and animal studies using tES are unlikely, our increased understanding on the underlying mechanisms of tES entrainment can guide researchers towards the best strategies that can drastically improve the reliability and validity of stimulation protocols^26^. For example, considering the differences in experimental setup and current densities between animal models and human studies, it is important to match the electric fields in the brains of different species instead of using the same stimulation parameters. New strategies for modeling electric fields that are built on the specific morphology of the animal brain and are not a projection from human models could greatly improve this effort. In addition to the practical considerations outlined above, this choice should be made thinking about the final purpose of each experiment. The design, type and dose of the stimulation should be always critically chosen and openly discussed in light of the chosen research question and it can—and should—change depending on whether tES is used as a tool to test or manipulate brain circuits, or if the goal is to evaluate the clinical efficacy of a specific tES protocol in a preclinical study.

### Comparable network effects

The mammalian brain can be parcellated into anatomically distinct areas, or modules, that are domain specific in function. In tES studies, it is usually one of these areas that is defined as “target” structure. However, complex behavior is rarely controlled by a single brain region. Instead it results from interactions between remote yet anatomically connected areas that form specialized circuits and networks. We have previously discussed how the magnitude of tES-induced effects on neuronal activity in a given region varies with the strength of the electric field. Yet, we argue that the consequences for behavior strongly depend on the effectiveness of tES to modulate local functional circuits and large-scale networks^10,47,48^. Given that disrupted information transfer across multiple brain regions (or networks) represents the basis for the theoretical description of several human brain disorders^49^, understanding direct consequences of tES or any other neuromodulatory intervention on network activity is a compelling area of ongoing research in human and animal models.

One of the key concepts when studying network effects with tES is that exogenously induced polarization fluctuations of an entire population of neurons is capable of providing a substrate for signal amplification^39^. It is important to note that the probability to react to an external electric field (i.e. the coupling constant) of a neuron embedded in a neural network can be different than that of an isolated neuron^50^. Due to the intrinsic synchronization of the neural network, a given neuronal unit can be polarized both directly from the field and indirectly from afferent neurons. The net effect of the stimulation results from the convolution of these two processes^35^. In general, stronger currents are required to obtain neural entrainment when the exogenous patterns compete with native brain rhythms of a neuronal network^31,37^. Strategies that facilitate this process exist, for example by tailoring the stimulation to specific and individualized brain frequencies and phase^51^. However, perfectly in-phase synchronicity may not always lead to increased functional coupling and network entrainment^52^ due to phase lags and neural transmission delays^35^.

Besides their use for testing the clinical feasibility of tES and exploring the mechanism of network entrainment, animal models could also become a tool to assess therapeutic usefulness of a given stimulation protocol. However, direct comparison of animal and human tES studies requires not only the proper calibration of stimulation parameters, but assumes the presence of homologous functional networks that govern the same behavioral processes across species. This assumption is studied in a novel neuroscientific field, comparative functional neuroanatomy, which examines the similarities and differences of the central nervous system from evolutionary perspective to dissect which neural networks underlie common behavioral patterns^53–56^. The results of recent investigations using this approach were able to define in which neural networks rodents and monkeys share a homologous organization as detected in humans, including the visual^54^, the salience^57^, the default-mode^58^ and the limbic networks^55^. This information is extremely valuable and can assist the design of tES studies and the clinical interpretation of electrophysiological and functional magnetic resonance (fMRI) recordings on animals in the context of large-scale networks modulation and entrainment.

**Fig. 2:**
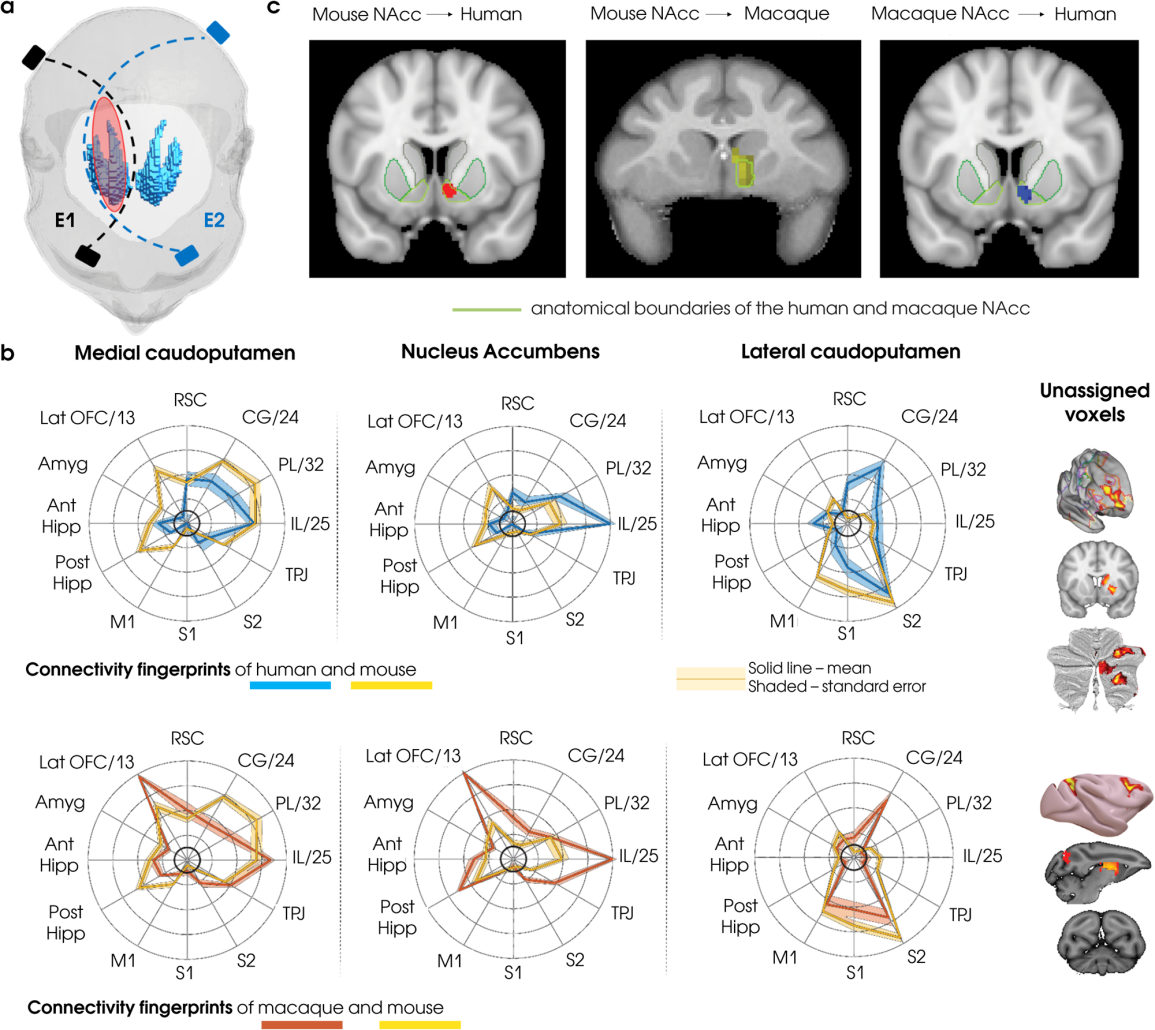
Comparison of striatal functional connectivity between mice, monkeys, and humans. The translation of the results acquired in the transcranial electrical stimulation (tES) experiments from animals to humans can be challenging due to anatomical and functional differences in the brains. However, it is possible to tackle this problem via comparative functional neuroanatomy approach. The utility of this approach was established in a recent study^55^ that compared patterns of striatal connectivity derived from resting state functional magnetic resonance imaging (rsfMRI) of mice, monkeys, and humans. First, researchers identified three clusters of connectivity patterns in the mouse striatum: medial caudoputamen, lateral caudoputamen, and nucleus accumbens (NAcc) and correlated their activity with 12 target regions known to be homologous across species. Afterwards, the connectivity fingerprints of all striatal voxels in human and macaque brains were compared with three connectivity patterns of a mouse. **a** Schematic depiction of the temporal interference (TI) stimulation reaching the right striatum in the human brain. **b** Connectivity fingerprints of human and macaque voxels that were significantly similar to the mouse cortico-striatal connectivity patterns. Most of the striatal voxels were unassigned as they were not significantly similar across species (85% for humans and 69% for macaque). Furthermore, some unassigned voxels were significantly different in comparison to the mouse fingerprints (26% of unassigned voxels for humans and 20% for macaque). The far-right columns of panel **b** present the regions that had higher connectivity with the unassigned voxels when compared against all three mouse connectivity patterns. Among these regions are prefrontal structures, which are more developed in primates than in rodents. **c** The smallest number of the unassigned voxels was found in NAcc, suggesting that the network of this region is preserved across species and it can be used as a target for the tES translational studies. Highlighted regions represent voxels of human (left) and macaque (middle) striatum that had statistically similar connectivity fingerprint with mouse NAcc. Human regions assigned to macaque NAcc are shown in the right image. Panels **b** and **c** are adapted from ref. ^55^, CC BY 4.0 (https://creativecommons.org/licenses/by/4.0/).

As an example applied to our previous argumentations, consider the following scenario: Imagine that a research team has optimized a tES protocol allowing to stimulate a deep cortical structure based on a method such as TI^14^, for instance the basal ganglia (BG) (Fig. 2a). Now assume that before rushing to directly apply this tES protocol in humans, the experimenters first test the neurophysiological plausibility of entrainment on a particular segment of the BG in an animal model, for instance in mice. Also, assume that the experimenters test the behavioral consequences of this protocol, finding that the intervention indeed induces the hypothesized behavioral change. Once the experimenters confirm that the protocol works in mice, the tES parameters are adapted for human application—presumably applied to the homologous brain area. Now the question is the following: In this scenario, should the investigators expect a reliable translation of the physiological and behavioral outcomes from mice to humans? Our answer is: It depends. A recent comparative functional neuroanatomy investigation found that while some subportions of the BG were consistently identified as a reliable target for translational neuroscience between mice, macaques, and humans (e.g., the nucleus accumbens (NAcc), Fig. 2b and c), most of the striatal regions were unique and unassigned for the macaque and human brain, in particular the caudate body, which had a connectivity pattern that significantly differed across species^55^. This suggests that apparent discrepancies in behavioral outcomes that might have been encountered in our hypothetical scenario might have been rooted on the fact that the tES intervention was actually targeting distinct functional networks across species.

## Evaluating the effects of tES across different levels of abstraction

In tES research, a lot of attention has been payed to the influence of the experimental scale (micro, meso, or macro) and the type of the studied system (in vitro, in vivo, ex vivo) alongside their corresponding neurophysiological mechanisms^36,59^. However, we argue that the scale and type of the system under study are not the only critical parameters to consider, and certainly not in isolation. Investigators should also consider what hypothesized computations, which are governed by neural oscillations^60^, are modulated by tES alongside their influence on behavior. Importantly, given that neural oscillatory patterns have been shown to be preserved across the species^38^, one could argue that the reconstruction of functional networks studied in one species could be in principle translated to another one. In this case, translation can be achieved by partially circumventing the necessity of finding the homologous anatomy-based networks, because the decisive criteria is not purely the anatomy, but the rhythm and its underlying function.

In an attempt to encapsulate these aspects, we take an inspiration from the representation of the functional levels of information systems proposed by David Marr^61^, which interestingly has also been instrumental to study other aspects of neuro-cognitive research^62^:

1. Computational level: what type of function is computed, what is the final goal of the system under study?
2. Algorithmic level: how is the function computed, what algorithm is used for this computation?
3. Implementation level: how is the algorithm implemented that leads to the observed behavior?

Inspired by this scheme, we propose that tES studies can be viewed as both the action and interaction of different levels of abstraction, which might in turn be decomposed in different sublevels depending on the type of system or organism that is studied (Fig. 3). Here, we do not attempt to use the exact definitions of information processing systems proposed by Marr on each level, but rather use them as a template for tES research on different levels of abstraction. In order to lay out these ideas, we start by using a bottom-up approach, that is, from implementation to computation.

**Fig. 3:**
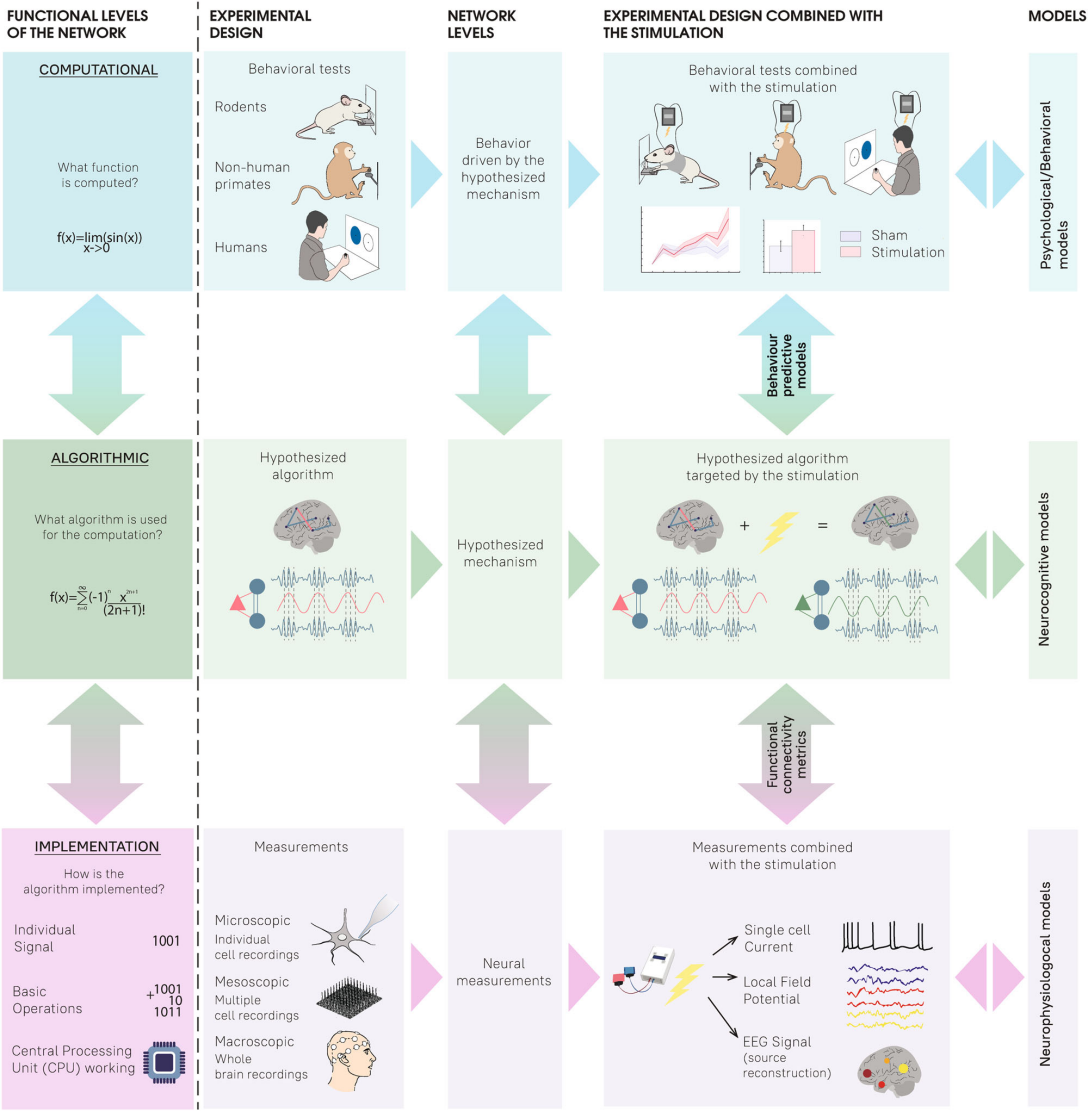
Studying transcranial electrical stimulation (tES) across different levels of abstraction. This figure conceptualizes the practical workflow and the effects of tES experiments. In order to encapsulate the study of tES-induced effects, we take inspiration from the Marrian levels of abstraction (“Functional levels of the network”) and adapt them to the tES experimental workflow (“Network levels”). The computational level reflects a computed function or organism’s behavior. The algorithmic level is assigned to a particular mechanism of the system under study. The implementation level refers to the physical or physiological changes occurring at the different scales of the studied system upon the realization of the algorithm. Color coding illustrates the assignment of tES experimental stages to a particular Marrian level, and the arrows highlight the transition between the levels. In neuroscience research, experimenters typically design behavioral tasks that should be carried out on a given organism (e.g., rodents, non-human primates, or human; “Behavioral tests”), whereas measurements of neural activity (“Measurements”) at different scales (micro, meso, and macro) provide information regarding the effects of task solving processes in the brain. Based on behavioral and neural data, researchers can hypothesize a mechanism (i.e. algorithm, “Hypothesized Algorithm”) that the system presumably uses to accomplish the task (e.g., an orchestrated communication between the different brain regions by the means of neural oscillations). The evaluation of tES-induced effects at each experimental stage provides an opportunity to establish a causal link between the resulting behavior (“Behavioral tests combined with the stimulation”) and the hypothesized algorithm (“Hypothesized algorithm targeted by the stimulation”). Additionally, concurrent measurements of the neural activity (“Measurements combined with the stimulation”) with a given tES protocol could also be used to refine or revise hypotheses and the effects of the stimulation at the different experimental scales. Computational models (“Models”) can be used to interpret the experimental results of tES experiments, as well as to refine the hypothesized algorithm (“Neurocognitive models”) and optimize experimental protocols (“Neurophysiological models” and “Psychological/Behavioral models”).

### Implementation level: neural measurements

We define this level as the units of the system that the experimenter can measure at different temporal and spatial scales: microscopic (e.g., single/multiunit recordings of spiking activity), mesoscopic (e.g., local field potentials (LFPs)) and macroscopic (e.g., electroencephalography (EEG), fMRI)^63^. With the application of a given tES protocol, researchers can assess the potential influence of the stimulation on these neural readouts.

Rhythmic changes in neural activity induced by tES can be quantified by the measurements of individual spiking activity (e.g., single unit activity^21^), the recording of an ensemble of single unit activities, or by the assessment of the neural population activity—via LFPs. At this level, periodic fluctuations of the electromagnetic field generated by the targeted neural population are assumed to be related to “functionally relevant brain rhythms”^21,36^. One of the potential action mechanisms of tES is the rhythmic alteration of the membrane potential of the neurons, causing the facilitation/inhibition of a specific neural oscillation in a target brain structure^35,36,59^. Crucially, the exact biophysical mechanisms governing the hypothesized neural entrainment induced by tES will depend not only on the parameters of the externally applied field (e.g., frequency, amplitude, direction of current flow, etc.), but also on the circuitry scale (e.g., slice, whole brain) and organism state (in vitro, in vivo, or ex vivo). For instance, evidence suggests that hippocampal–cortical interactions affect cortical endogenous activity that, crucially, depends on the status of an organism: sleep or awake^64^. Therefore, when researchers study isolated cortical slices or different behavioral states of an organism (sleep or awake), such distinct scenarios will inevitably affect the fundamental mechanisms governing tES-induced effects, such as: stochastic resonance, rhythm resonance, temporal biasing, network entrainment, and imposed pattern^36^.

Nevertheless, it is important to emphasize that the selection of the specific organism, state, and circuitry scale allows the researchers to study the broad spectrum of physiological effects of the applied tES protocol on neural oscillations (Fig. 3). For instance, in vitro slice studies^50,65^ and in vivo research in rodents^21,28^, macaques^22,66^ and humans^37,67^ offer the possibility to connect physiological effects (e.g., ephaptic coupling^65^, neuronal excitability^28^, neuronal entrainment^21,22,28,50,66^, spike timing^22,50^, synaptic plasticity^67^, potential peripheral stimulation^68^) caused by the applied stimulation protocol with the parameters of the applied field: amplitude, frequency, directionality. Once the physiological effects of the applied tES have been delineated, researchers can use this knowledge to reveal the influences of these modulations at the network level.

### Algorithmic level: Hypothesized mechanisms

At this level, the experimenter proposes a formalized model of oscillatory network operations driving the observed behavior. Therefore, researchers have the opportunity to hypothesize what algorithm is implemented through neural oscillations that facilitates efficient communication mechanisms in neural networks (examples include communication through coherence^69^ and cross-frequency coupling^70^). One group of methods assessing the interplay between neural oscillations include “connectivity-based” measures of statistical dependencies such as: phase-locking, coherence, correlations, etc.^1^. An alternative approach comes from the application of information theoretical concepts, such as mutual information (MI) and transfer entropy (TE)^71^, which estimate the transfer of predictive information between two or more time series^72^. Since transfer of predictive information reflects the actual computation conducted by the network (not its synchrony or coupling strength^71^), relationships found via interactions between the neural oscillations from different brain regions are hypothesized to reflect a reciprocal information transfer caused by computations performed at different nodes of the brain. Therefore, the use of approaches based on MI or TE may serve to detect computation-related information transfer, potentially separating it from the information transfer driven solely by the anatomy and biophysics of the studied system^71^. Specifically, TE together with active information storage and local information modification^71^ allows the researcher to assess the computational processes, whereas the metrics for causal interaction (e.g., causal information flow^73^) reveal the set of all possible causal interactions of a system^71^. Thus, the comparison of these two families of metrics potentially allows the scientists to distinguish between the computational and anatomy-driven information flows.

While testing whether a given tES protocol induces modulations in specific latent variables of the hypothesized mechanism, it results essential to test whether these algorithmic changes are reflected in corresponding modulations of behavior.

### Computational level: functional changes

At this level, the experimenter quantifies what aspects of observed behavior in a given task (e.g., hit rates, decision accuracies, reaction times, etc.) or cognitive function (assessed via computational cognitive models^37^) are related to the hypothesized mechanism at the algorithmic level. Here, tES provides the opportunity to interfere with the hypothesized mechanism, enabling to establish a causal relationship between the physiological mechanism and behavior. Unfortunately, given the difficulties of concurrent tES and electrophysiological measurements using non-invasive brain recordings methods^74^, the classical approach is to directly assess the behavioral impact of the applied tES protocol (e.g., real stimulation vs. sham, or the relationship between a given tES-induced oscillation phase and behavior^8,75^). However, we argue that the tES field of research requires parsimonious and accurate statistical inference methods for joint models of neural and behavioral measures. Typically, researchers adopt the so-called “two-stage” correlation approach where parameters of a fitted cognitive model are simply correlated with the neural measure of interest. However, these approaches do not enforce a constraint on the model parameters based on random variation in neural data. Here we argue that it is precisely this lack of constraint that makes it difficult to establish a connection between the computational and algorithmic level in our proposed scheme (Fig. 3).

Recent developments in statistical models of cognition and behavior propose a better integrative approach to study brain–behavior relations, the “joint modeling” approach^76^. In brief, the joint modeling approach consists of two parts:

1. A behavioral model that is used to capture the behavioral observations. For example, in a perceptual detection task one could opt to use signal detection theory (SDT) models to quantify hit and false alarm rates^77^. Another example could be the use of the popular drift diffusion model (DDM) to capture choices and reaction times in a decision-making task^78^. The goal of the experimenter is usually to find the set of latent variables in each model that more closely predicts the observed behavioral data.
2. A neural model that relies on the physiological data to estimate a neural metric of interest associated to the current behavior. For instance, one could obtain trial-by-trial fluctuations of a given functional coupling metric based on EEG recordings^79^.

Here, it is important to emphasize that on the one hand, the classical approach relies on two-step correlation analyses (i.e., brain data and behavioral data are treated as “independent” sources). This approach is not ideal given that non-invasive brain stimulation studies typically rely on rather limited sample sizes and second level correlations in small samples are likely to make type I errors. On the other hand, the joint modeling approach treats neural data as covariate of the behavioral data. This enforces a constraint on the behavioral model parameters based on the random variation in the neural data, where inference is based on the richness of trial-wise information. Thus, risk of type I errors is minimized. For the specific case of brain stimulation applications, one could then quantify how such relationships are modulated by a given stimulation condition on a trial-by-trial basis (e.g., sham vs. real). More detailed and technical information about the specification of joint models can be found in ref. ^76^. Taken together, we believe that adopting more precise quantitative models at the different levels of analyses (see “Models” in Fig. 3) could lead to deeper and more precise understanding about the feasibility of tES as a tool to study the causal role of neural oscillations on cognition and behavior.

## Towards individualized tES interventions

The ultimate goal of tES experiments is to generate stable and foreseeable neurophysiological and behavioral effects in every volunteer or patient. However, to date, the effects of electrical stimulation are not reliably observed at the individual level^26,80^. Additionally, neurophysiological responses to both sensory processes and tES-induced modulations can be highly variable across individuals^81^. These observations have generated an increasing interest in the development of realistic head models and methodological solutions that consider individual morphometry and neurophysiological responses. We argue that these approaches will play a fundamental role in the optimization of stimulation parameters and their underlying neurophysiological and resulting behavioral effects.

### The importance of predictive models

The generation of individual head models starts with the assessment of the subject’s structural magnetic resonance imaging (MRI) scan that is segmented into several, typically six tissue types, including the scalp, skull, CSF, gray matter, white matter, and air^82^. Afterwards, conductivity values associated with these tissues are assigned, then stimulation electrodes ascribed to the peak intensity of the current are placed on the head, and finally, the electric field distribution in the brain is calculated with the finite-element method (FEM). This procedure allows estimating the strength of the electric field, evaluating the dose of the stimulation delivered to the brain, and examining the spatial distribution of the electric field to estimate the focality of the stimulation.

Only recently, predictions of the electric field strength and distribution derived from individual head models were compared and validated with intracranial recordings in vivo in humans^83^. The distribution of the electric field was found to be well predicted by head models, meaning that individual simulations can provide valuable information for the comparison of the impact of the brain stimulation on the region of interest and other brain areas^31,84^. Furthermore, based on the spatial distribution of the electric field researchers have an opportunity to compare different electrode configurations and to select target region of interest in the most efficient way. Nevertheless, it is still debated, whether results of individualized models can be used to predict current doses for each participant to reach the same strength of the electric fields across individuals^85^. When model predictions were compared to the invasive recordings, the difference in the absolute field strength between these estimations reached 40%^84^. Furthermore, the sign of this error cannot be reliably predicted given that the strength of the electric fields can be underestimated and overestimated by the models. Although this remains an unresolved issue, estimation of the absolute electric fields with individual models can be still considered as a useful tool for planning tES experiments^86,87^ (we expand on this matter below). While the effect of stimulation depends on both structural and functional connectivity^88,89^, introduction of the latter in standard pipelines for individual models may improve predictions of the electric fields^90^.

In the last years, several studies have demonstrated that introduction of individualized head models can explain some of the variability in neurophysiological signal readouts^87,91^. Compelling evidence of this sort comes from a recent study, where researchers attempted to predict the impact of tACS on the power of alpha oscillation recorded with magnetoencephalography (MEG)^87^. The investigators built individual electric field models to estimate its strength and the spatial precision of stimulation, which corresponded to the correlation between the spatial distribution of the electric field and the alpha topography. Incorporation of these variables allowed the model to predict from 51% to 87% of variance of the individual alpha power modulations induced by tES, supporting the idea that individual modeling can predict the neurophysiological effect of the stimulation. In other studies, simulations of the electric fields at the individual level allowed to predict resting-state fMRI (rsfMRI) modulation of brain networks^91,92^ and motor-evoked potentials^86^, thus providing further evidence for the usefulness of individualized head models.

### Other approaches for individualized interventions

One of the ultimate goals in tES research is to develop effective and reliable interventions for neuropathological conditions. However, it is essential to consider that in such conditions brain anatomy typically differs from those of healthy individuals^93^. This problem could be partially solved with the incorporation of individualized head models that take into account between-subject variability in anatomy and therefore guide researchers to employ more effective stimulation parameters. The majority of efforts for individualizing tES interventions typically focus on applications for healthy brains, but fortunately this problem is starting to be addressed in the field for the case of neuropathological conditions^89,94^. Nevertheless, establishing first how to tackle inter-individual variability and the effectiveness of tES interventions in the healthy brain will be essential to inform and adjust predictive models and stimulation protocols in pathological conditions.

State-of-the-art models cannot be adjusted to the dynamic nature of oscillations that vary within participants over time. To resolve this problem, researchers are starting to develop closed-loop systems, which can be used to adjust the parameters of stimulation to the frequency and the phase of the individual neural oscillation. However, to date this technique is not fully reliable for conditions, when frequency of tES matches the neural oscillations that is investigated, due to the severe artifacts in the recordings elicited by the stimulation^74,95^. Considering that this is a common scenario, researchers explored several ways to overcome this limitation. One approach is to conduct MEG and EEG recordings before the stimulation session and then adjust tACS intervention frequencies to the correspondent individual’s frequency^87,96^. A second alternative is to match both the frequency and the phase of the stimulation to the brain oscillation with intermittent recordings that are collected during the intervals when the stimulation is switched off^9^. A third alternative is based on simultaneous sensory and electrical stimulation^75^. However, this methodology might be limited to low-level perceptual domains, where delays in the propagation of the percepts must be considered. Another alternative is to generate a non-conventional tACS current waveform, e.g., sawtooth, allowing the possibility of artifact removal from the EEG signals^97^.

An additional source of variability is conductivity of the head tissues which changes within and between participants and is vital for the accurate estimation of stimulation currents delivered to the brain^31^. More accurate conductivity values at the individual level can be acquired with the development of non-invasive imaging methods such as electrical impedance tomography (EIT), or MRI and diffusion tensor imaging (DTI)^98^. A new emerging technique, MRI current density, could also be used to estimate both conductivities and the current flow on a personalized basis^99^. Overall, we argue that further development of stimulation protocols that incorporate anatomical and physiological details between and within subjects will increase the efficacy of tES interventions for therapeutic purposes.

## Conclusions

In the last five decades, a vast amount of research strongly points to the fact that in humans and other animals neural oscillations are an essential mechanism to efficiently transmit information in the brain for guiding behavioral and cognitive processes. However, causal manipulations in humans await approval. Critically, this limitation is particularly relevant in clinical settings given the observation that various neurological and neuropsychiatric disorders are linked to abnormal neural oscillatory dynamics^100^. It is only in the last decade that tES methods have been proposed as indispensable tools for elucidating how behavior may causally depend on brain rhythms in the intact human brain. However, current controversies leave unclear whether tES methodologies can indeed be used to test the causal role of neural oscillations in a safe and effective manner in humans. Crucially, the amount of critical knowledge that has emerged in the last 5 years is the result of a much-needed paradigm shift into an integrative research agenda. While the research carried out at each level of description provides essential knowledge about the mechanisms and efficacy of tES methods to induce neural entrainment, we emphasize that care should be taken before jumping into conclusions on whether the presence or absence of effects observed in a given species or behavioral state can be safely translated to other domains. We also argue that combination of practical experimental data with theoretical modeling estimations may improve not only the translation of the results across species, but also reduce the variability in response to tES especially when combined with approaches that allow to adjust the stimulation parameters on the individual basis. We foresee that information provided from coordinated methodological efforts to optimize the conclusiveness of findings on relations between neural oscillations and behavior will become increasingly important for assessing the translation potential of tES protocols from animal models to humans. However, in order to predict more accurately the clinical outcome of these interventions in humans, a good agreement must exist in the mechanisms of action at the network and behavioral levels across species and behavioral states. These integrative efforts will be vital for successful translational applications of tES methodologies to improve mental health.
